# Uncertainties Involved in the Use of Thresholds for the Detection of Water Bodies in Multitemporal Analysis from Landsat-8 and Sentinel-2 Images

**DOI:** 10.3390/s21227494

**Published:** 2021-11-11

**Authors:** Luis Gustavo de Moura Reis, Wendson de Oliveira Souza, Alfredo Ribeiro Neto, Carlos Ruberto Fragoso, Antonio Miguel Ruiz-Armenteros, Jaime Joaquim da Silva Pereira Cabral, Suzana Maria Gico Lima Montenegro

**Affiliations:** 1 Center for Technology and Geosciences, Federal University of Pernambuco (UFPE), Recife 50670-901, Brazil; luisgustavo.mr@gmail.com (L.G.d.M.R.); alfredo.ribeiro@ufpe.br (A.R.N.); jaime.cabral@ufpe.br (J.J.d.S.P.C.); suzanam.ufpe@gmail.com (S.M.G.L.M.); 2 Center for Technology, Federal University of Piauí (UFPI), Teresina 64049-550, Brazil; wendsonsouza@ufpi.edu.br; 3 Center for Technology, Federal University of Alagoas (UFAL), Maceió 57072-970, Brazil; 4 Department of Cartographic, Geodetic and Photogrammetry Engineering, University of Jaén, Campus Las Lagunillas, s/n, 23071 Jaén, Spain; amruiz@ujaen.es; 5 Microgeodesia Jaén Research Group (PAIDI RNM-282), University of Jaén, Campus Las Lagunillas, s/n, 23071 Jaén, Spain; 6 Center for Advanced Studies on Earth Sciences, Energy and Environment CEACTEMA, University of Jaén, Campus Las Lagunillas, s/n, 23071 Jaén, Spain

**Keywords:** MNDWI, reservoir, remote sensing, water level tracking, Poço da Cruz reservoir, water spectral index, caatinga biome

## Abstract

Although the single threshold is still considered a suitable and easy-to-do technique to extract water features in spatiotemporal analysis, it leads to unavoidable errors. This paper uses an enumerative search to optimize thresholds over satellite-derived modified normalized difference water index (MNDWI). We employed a cross-validation approach and treated accuracy as a random variable in order to: (a) investigate uncertainty related to its application; (b) estimate non-optimistic errors involving single thresholding; (c) investigate the main factors that affect the accuracy’s model, and (d) compare satellite sensors performance. We also used a high-resolution digital elevation model to extract water elevations values, making it possible to remove topographic effects and estimate non-optimistic errors exclusively from orbital imagery. Our findings evidenced that there is a region where thresholds values can vary without causing accuracy loss. Moreover, by constraining thresholds variation between these limits, accuracy is dramatically improved and outperformed the Otsu method. Finally, the number of scenes employed to optimize a single threshold drastically affects the accuracy, being not appropriate using a single scene once it leads to overfitted threshold values. More than three scenes are recommended.

## 1. Introduction

Several studies have been dedicated to enhancing water features extraction by remote sensing techniques aiming to monitor and characterize hydrological dynamics of lakes and reservoirs [1,2,3,4,5,6].

The accuracy in water feature extractions by remote sensing has been constantly pursued by the development of several methods and approaches, such as: enhancement of water spectral indices [7,8,9,10,11]; use of supervised classification algorithms based on machine learning [12] to segment water/non-water features and tailor an appropriated threshold [13] and combining different bands, sensors and spectral indices [14,15,16,17].

Threshold segmentation remains attractive due to its easy implementation, lower investment of time, and relative accuracy in extracting water features [8,13]. However, selecting an appropriate threshold to maximize accuracy is challenging and time-consuming [7], and can underestimate or overestimate surface water features due to its spatial-temporal variability [17].

Despite the well-known limitation of employing a single threshold to correctly classify all instances, especially in spatiotemporal classification problems [13,18], the results from Bangira [12] and Sarp and Ozcelik [19] evidence that the performance of supervised classification methods is slightly superior to those achieved through threshold segmentation which still puts its use as a solution feasible to be implemented producing accurate results.

In addition, factors such as complexity land-covers near water boundary, backgrounding effects, physical-chemical and biological water characteristics (e.g., turbidity, color, chlorophyll), presence of aquatic vegetation, shadows, mixed pixels, coarse pixel resolution affect negatively more sophisticated classifying methods as well [7,12], therefore, not being an exclusive limitation related to threshold methods.

On the one hand, several works recurrently point out that a single global optimum threshold approach is a suitable technique for time series analysis and track water dynamics [8,13]. On the other hand, the accuracy variability from its application is understudied. So far, there is no knowledge of research in which accuracy’s variability, calculated over water level estimation using Landsat-8 and Sentinel-2 imagery, has been used to support the choice of the threshold. Furthermore, the accuracy exclusively from orbital imagery, not considering errors due to coarse digital elevation model resolution, has not yet been investigated.

Apart from that, most optimization approaches are based on the premise that the lack of threshold stability may be a problem [18], which leads to a subjective choice [7]. A stable optimal threshold value has been regarded by many authors as an indication that a single threshold value is suitable to classify multiple scenes across space and time [20]. Conversely, results from Du [21] and Weekley and Li [1] indicate that the accuracy stability could be reached even in a threshold range variation, depending on the index and methodology used.

The purpose of this paper is to investigate the uncertainty involving using a single optimized threshold over multitemporal Landsat-8 and Sentinel-2 imagery scenes. Our primary objectives were: (1) understanding how accuracy varies with thresholds values to identify ranges less subjected to errors; (2) investigate accuracy statistics, exclusively from orbital imagery through employing a high-resolution digital elevation model, and to compare each sensor performance; (3) comparing the advantage in using the proposed cross-validation approach to support threshold choice versus a nonparametric and unsupervised method of automatic threshold selection; and (4) contributing to operational steps improvement in adjusting a single threshold in multitemporal classification problems, regarding the accuracy and computational effort.

## 2. Materials and Methods

### 2.1. Study Area

**Figure 1:**
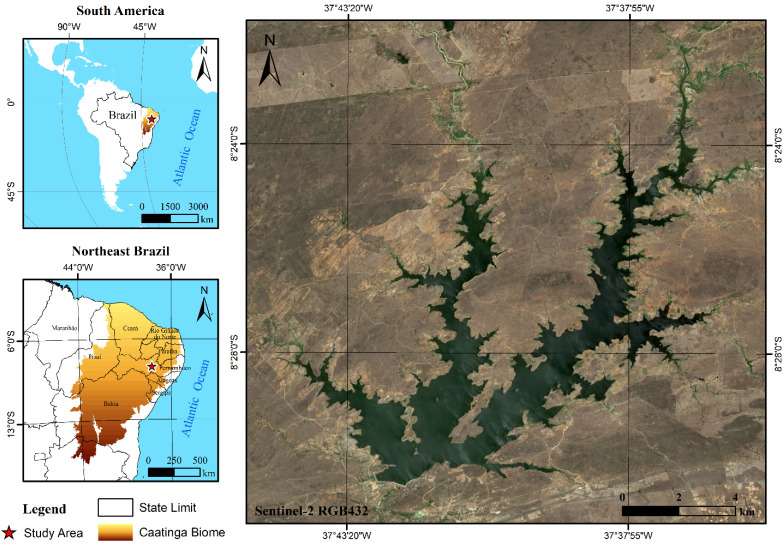
Location map of the test area.

The ‘Poço da Cruz’ (PC) reservoir, in Figure 1, was commissioned in 1958 and is located on the Moxotó River, an intermittent tributary of the São Francisco River, one of the most important rivers basins in Brazil. According to recent bathymetric surveys, its storage capacity is 0.484 km^3^, corresponding to a maximum surface area of 56.34 km^2^. The Moxotó River Basin drains about 9619 km^2^, and the area affluent to the reservoir is 4716 km^2^. The region’s climate is semi-arid, where the mean annual rainfall is 421.8 mm, highly concentrated during the four months from December to May. Evaporation rates are about 1568 mm/year. The study area is inserted at the Caatinga biome, a seasonal tropical dry forest present in South America, characterized by a high albedo during the dry season and vegetation health highly correlated to precipitation and soil moisture.

### 2.2. Data-Sets

#### 2.2.1. Digital Elevation Model

The digital elevation model (DEM) of the PC reservoir was provided by the Brazilian Water Agency (ANA) and consisted of an aerial laser survey using the LiDAR (Light Detection and Ranging) method for the land part integrated with a bathymetric survey for the flooded area. The latter was carried out with an eco-probe using a single beam for shallow waters and a multibeam for deep waters. Both were referenced to the Brazilian Geodetic System (SGB), resulting in a density DEM of 2 points/m^2^ and a 1 m resolution image [22].

#### 2.2.2. Data Acquirement and Image Pre-Processing

The image acquisition and pre-processing were carried out using the Google Earth Engine (GEE), a platform for geospatial cloud processing that makes available several spatial Earth-imaging missions and collections [23]. Satellite imagery was selected and processed through this tool to generate the water Modified Normalized Difference Water Index (MNDWI) images. This index proposed by Xu [11] introduced the short-wave infrared (SWIR) rather than the NIR band [10]. Such modification enhanced: (1) the ability to suppress vegetation and built-up noise; (2) the stability of the adjusted thresholds [11,18]; and (3) the accuracy in classifying impure pixels [14]. Furthermore, MNDWI is one of the most widely used water indices in surface water mapping, providing a great empirical application base.

MNDWI images were composited from Landsat-8/OLI (Operational Land Imager) and Sentinel-2/MSI (Multispectral Instrument) using the following dataset available in GEE:

- Landsat-8 Surface Reflectance Tier 1 collection provided by United States Geological Survey (USGS), a Level-1 precision and terrain corrected product, atmospherically corrected surface reflectance using LaSRC algorithm, with a 30 m resolution, 16-day temporal resolution. Bands B3 (green) and B6 (SWIR) were used in the MNDWI composition. We used 19 free-of-cloud images available from 14 April 2013 to 24 September 2020;
- Sentinel-2 collection provided by the European Space Agency (ESA), a Level-2A orthorectified product, atmospherically corrected surface reflectance using the Sen2Cor algorithm. Along with a 5-day temporal resolution, SWIR band (B11) available with 20 m spatial resolution, and Green band (B3) available with the 10 m and resampled to 20 m were used in the MNDWI composition. We used 24 free-of-cloud images available from 22 December 2018 to 17 September 2020.

#### 2.2.3. Hydrological Monitoring Data

The observed water levels (OWL) from the PC reservoir were provided by Waters and Climate Agency of Pernambuco state (APAC), available in a daily temporal resolution, starting on 1 July 2000. In addition, OWL was converted to the SGB by adding 0.9 m. Gaps in OWL corresponding to the image acquisition dates were filled using linear interpolation according to Table 1.

### 2.3. Threshold Optimization and Uncertainty Analysis

#### 2.3.1. Image Segmentation and Water Level Estimation

**Figure 2:**
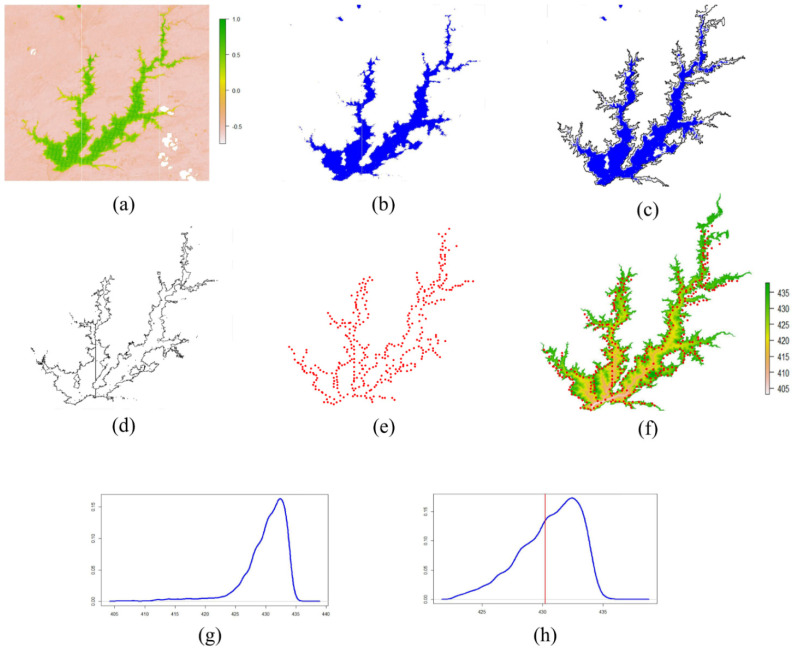
Water level estimation process: (**a**) MNDWI images; (**b**) thresholding MNDWI images; (**c**) masking MNDWI images by a buffer derived from DEM, considering the maximum elevation at 437 m (2 m higher than the maximum operational limits) to eliminate disconnected water bodies and WL values arising from misclassified pixels; (**d**) creating a polygon from raster MNDWI masked image; (**e**) converting polygons to border points; (**f**) extracting elevations at point positions from DEM; (**g**) removing outliers from extracted elevation dataset; (**h**) water level estimation (*Yc*) from the median statistic.

The image segmentation and water level (WL) estimation process are illustrated in Figure 2. All geospatial operations necessary to image segmentation and water extraction elevation (*Yc*) were automated by using the following R packages: sf [24] and raster [25]. In addition, data manipulation and statistical analysis were also conducted in R, using the packages: dplyr [26], ggplot2 [27], and reshape2 [28].

First, the predefined threshold T was used to segment all MNDWI images in the dataset (Figure 2a), whereby values greater than T were classified as water (Figure 2b). After water/non-water segmentation, a mask derived from DEM, corresponding to the maximum elevation at 437 m (2 m higher than the maximum operational limits), was applied over the binary classified raster layer to discard pixels outside its boundaries (Figure 2c).

This geospatial operation was carried out to avoid WL arising from misclassified pixels and disconnected water bodies. Similar technics were used by [29] to constrain the study area and exclude effects from build-up areas, anthropogenic land cover, and buildings. After masking, the raster was converted to polygon (Figure 2d), and then vertices transformed to points (Figure 2e). A WL dataset was extracted from DEM at coordinate points (Figure 2f).

After DEM extraction, a large dataset of WL values is generated as output, being necessary to choose a unique representative WL (*Yc*) value to calculate accuracy (Equation (2)). This problem is circumvented by adopting the Median statistic, which represents a central tendency of data.

Before the Median calculation, the Tukey method [30] was applied to remove extreme values from the extracted WL dataset (Figure 2g). This procedure reinforces noise reduction and removes undesirable extreme WL values, such as remaining misclassified pixels at higher land and border points identified inside the water body (arising from image failures such as vertical lines Figure 2a,d,e).

In addition, some characteristics present in PC inlet branches, such as agriculture, shallow waters, pastures, macrophyte vegetation, and eutrophication, have a substantial similarity of wetlands, marshland, and inundated areas. Hence, it cannot be correctly addressed without a sub-pixel level approach [31], which leads to omission errors in water detection [32]. WL extracted from these areas probably corresponds to Figure 2g lower tail and were effectively trimmed after filtering (Figure 2h).

Therefore, the values beyond the higher and lower limits shown in Equation (1) are removed to aggregate more confidence and stability to extracted WL dataset. The variables Q_1_ and Q_3_ represent the first and third quartiles from extracted WL values, respectively. These steps are illustrated in Figure 2g,h.

(1) $$Lower=Q_{1}-1.5\times \left(Q_{3}-Q_{1}\right)\;Higher=Q_{3}+1.5\times \left(Q_{3}-Q_{1}\right)$$

#### 2.3.2. Threshold Optimization and Accuracy Evaluation

**Figure 3:**
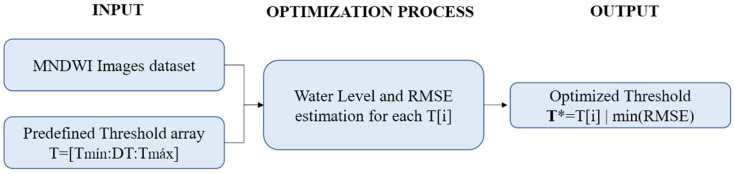
The optimization process ran over a selected images dataset and a predefined threshold array to find T*, which minimizes RMSE.

The general threshold optimization process is illustrated in Figure 3. It consists of an enumerative search, in which all the MNDWI images in the input dataset are segmented using the same single threshold value. The process illustrated in Figure 2 is carried out for each threshold T[i] in the predefined threshold array—which varies between a minimum and maximum value with an increment of 0.01. Some previous simulations were run to define the range of the threshold array aiming to minimize the computational effort.

By running the Figure 2 process recursively, each image in the input dataset has the corresponding estimated WL (*Yc*), which in turn is compared to its corresponding observed or filled water level (*Yo*), shown in Section 2.2.3, to calculate RMSE (Equation (2)).

Finally, the optimum threshold value T* is taken as the one corresponding to the minimum RMSE value after the enumerative search described above.

(2) $$RMSE=\sqrt{\frac{\sum_{i=1}^{n}{\left(Y_{C}-Y_{o}\right)}^{2}}{n}}$$

#### 2.3.3. Uncertainty Analysis

This work aimed to investigate uncertainties concerning using the single threshold approach. Moreover, to address a method to fulfill this purpose, we must consider that:

The single threshold method is a stump, a machine learning model consisting of a one-level decision tree, therefore an adaptative non-linear technique;

- Our interest is to support constructing a model capable of predicting WL on independent test data;
- A fitting method typically adapts to training data, and hence the training error is commonly an optimistic estimate of test error (generalization error) [33];
- Once each image produces only one WL (*Yc*) to compare with each observed gauges (*Yc*), our training set was limited to the number of Landsat-8 and Sentinel-2 scenes available (Section 2.2.2);
- With a restricted number of training instances (19 Landsat 8 and 24 Sentinel 2 images), we are not in a data-rich situation to divide the training and a test set;
- The computational effort required to tune our model (enumerative search) is considerable due to the complexity of the WL estimative process described in Figure 2;

The Bootstrap methodology [34,35] is the most suitable approach to be applied. By mimicking the cross-validation method, this method fits the model on a set of bootstrap samples drawn with replacement from the original dataset and then evaluates how well it predicts the original training set [33]. Given the nature of our optimization problem (Figure 3) that demands all the geospatial operations described in Figure 2 recursively, training and test were carried out as distinct processes, making it impossible to keep track of predictions not containing information used in threshold optimization.

Considering the computational burden of the optimization by enumerative search, 20 thresholds values (T*) were generated for each image dataset. The number of MNDWI images (*n*) taken as input in the training set of the optimization process (Figure 3) varied and assumed the predefined values *n* = (1,3,7) to investigate how this factor affects accuracy. Therefore, 6 datasets of 20 optimized thresholds (T*) were obtained: 3 MNDWI Landsat-8 and 3 MNDWI Sentinel-2.

**Figure 4:**
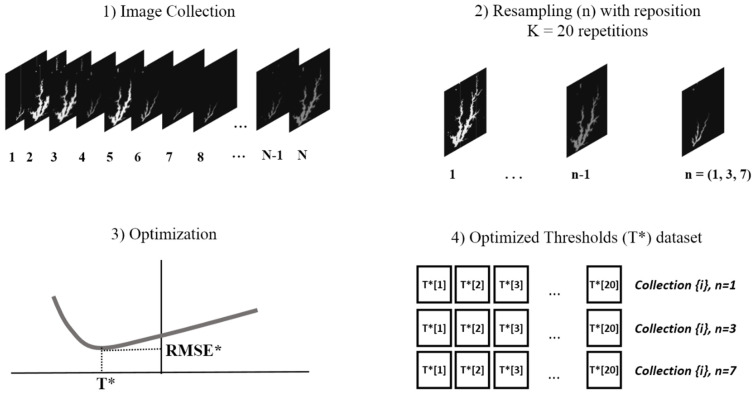
Optimized thresholds generation: (1) The original Landsat-8 (N = 19) and Sentinel-2 (N = 24) images data is assumed to be the population; (2) *n* = (1,3,7) images are drawn randomly from the population with reposition; (3) the optimization process described above and illustrated in Figure 3 is run to obtain T* as output, and the sampled images in the former step as input; (4) the process is repeated 20 times for each image collection and each sample size *n* = (1,3,7).

The input dataset images were randomly selected, with repositioning. In this approach, the whole dataset is assumed to be the population of size N (Figure 4) following the Bootstrap methodology mentioned above.

**Figure 5:**
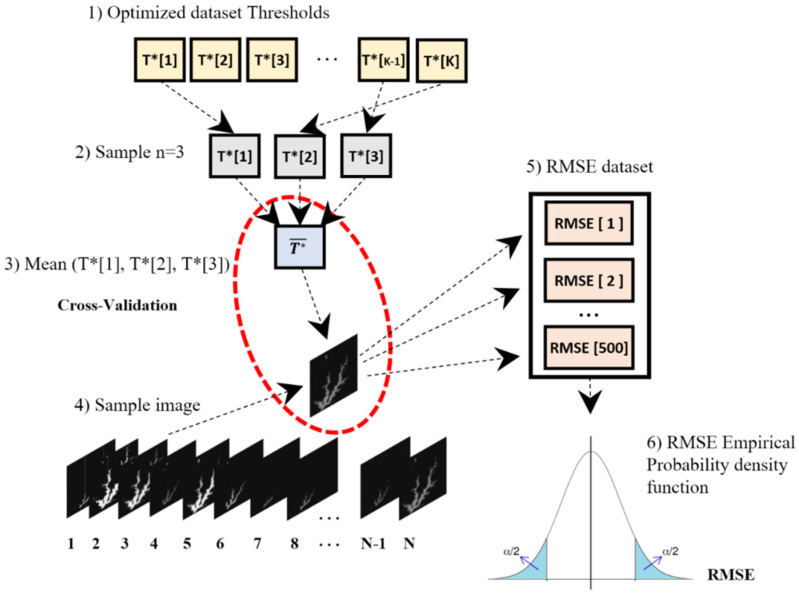
Estimation of RMSE empirical probability density functions of the from mimicked cross-validation: (1) Each dataset collection of optimized thresholds (T*) is supposed to be the population; (2) three elements of T* drawn by chance with reposition; (3) a mean optimized threshold value (${\bar{T}}^{*}$) is calculated; (4) a single image is chosen by chance, with reposition, from the original MNDWI Sentinel-2 or Landsat-8 dataset; (5) the process detailed in Figure 2 is carried out, the water level is estimated and compared to observed or interpolated, and RMSE is calculated; (6) steps 1 to 5 are repeated 500 times, the pairs [${\bar{T}}^{*}$, RMSE] are stored for accuracy’s statistical analysis.

The accuracy empirical probability density function was derived by employing the flowchart illustrated in Figure 5, which generated a dataset of 500 RMSE and T* values. It then was used to analyze the uncertainties concerning thresholding.

#### 2.3.4. Threshold Stability Analysis

The RMSE and T* datasets, generated by mimicked cross-validation, were analyzed to investigate intervals a ≤ T* ≤ b where the threshold selection leads to minimal and more stable RMSE values.

The OTSU algorithm, an automatic, unsupervised method [32], was applied to compare cross-validation results. The EBImage R package [36] was used to segment each MNDWI image, by maximizing the intraclass water/non-water pixel variance. Before OTSU segmentation, the images were masked to constrain the study area (described in Section 2.3.1).

## 3. Results and Discussion

### 3.1. Errors Involving Single Threshold

Errors involving thresholding arises from two main reasons:

- Thresholds values are not constant in space and time and vary due to subpixel water-land-cover composition [18] and due to environmental optical complexity [7] that affects reflected spectral profile such as biological water characteristics, presence of aquatic vegetation, and complex land-covers near water boundary;
- The noise arising from misclassified non-water features, whose spectral index values are similar to water [7,11,37].

**Figure 6:**
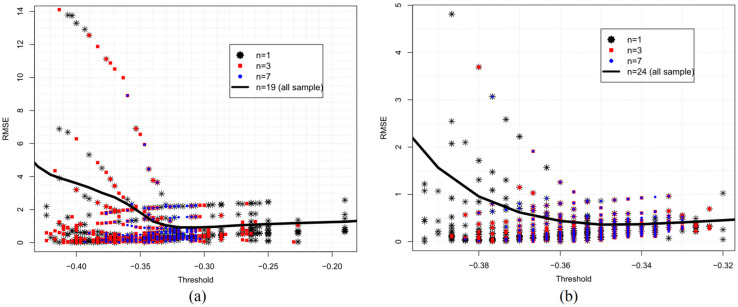
Scatter plot errors obtained from mimicked cross-validation: (**a**) Landsat-8 and (**b**) Sentinel-2 images.

Figure 6 suggests that the lower RMSE values represent errors from spatiotemporal reflectance variability, forming a block spreading randomly across the threshold axis. Visually, in Landsat-8 imagery, this type of error is more concentrated in RMSE values below 2.5 m (Figure 6a), while in Sentinel-2, it is concentrated below 1 m (Figure 6b).

Figure 6 also illustrates that noise from misclassification corresponds to higher RMSE values concentrated on the left side (lower thresholds). These errors are associated with higher negative MNDWI values, represented by pixels with high reflectance in the SWIR band, such as build-up, rock, roads, sand [18], and low albedo features, such as asphalt, shadows, mountains, building, and clouds [7].

### 3.2. Accuracy, Stability, and Precision

**Figure 7:**
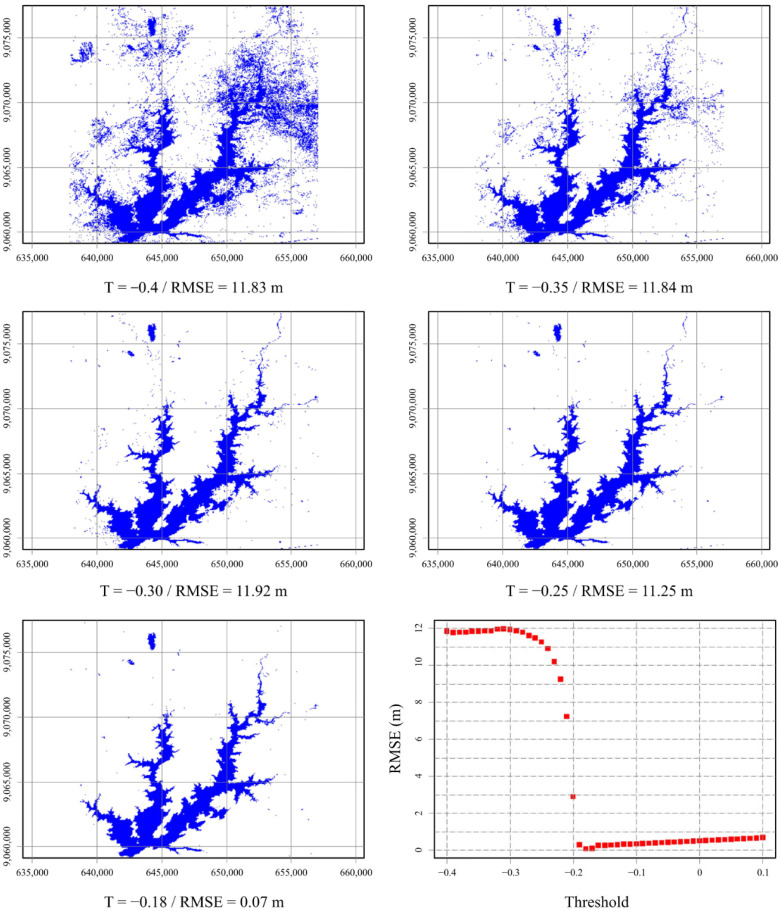
Threshold’s variation effect in water pixel classification noise.

As illustrated in Figure 6, errors arising from pixels reflectance variability are more limited, predictable, however unavoidable. Conversely, errors arising from noisy misclassification are higher, spread, and unstable. In this work, we removed noise effects through (a) masking MNDWI images and constraining the area (b) applying outlier filters, and (c) using the median statistic of extracted water levels. However, as illustrated in Figure 7, as threshold values are lower than the optimum threshold value (T*), noise drastically negatively impacts accuracy. These results follow Yang [37], who found that when the threshold is lower than a specific value, it brings too much noise that cannot be effectively eliminated, even by a filter.

Therefore, the optimized threshold T* can also be considered a critical value that leads to an unstable region in terms of accuracy. Aiming to minimize RMSE, the optimization process may take thresholds to highly negative values, which can work only in the specific scene and produces too much noise when applied in another one, affecting accuracy negatively. Hence, it is desirable to choose threshold values higher than T*, where error variations are lower, increase linearly, smoothly, and are more stable.

The cross-validation approach allowed to estimate the cross-effects of optimized threshold values (T*) over imagery dataset samples and thus to identify the stability region. However, as shown in Figure 6, the stability region can be identified using all imagery datasets (represented by the thick black line) without using a cross-validation approach, which requires less computational effort and is less time-consuming.

The optimized thresholds’ statistics illustrated in Table 2 shows that the mean values stay very close to the lower limit of the stability region. In terms of standard deviation (SD), the upper limits for Landsat-8 and Sentinel-2 can be reached by adding the mean value by a multiplication factor, 1.39xSD and 1.5xSD, respectively. This procedure was used by [29] to adjust threshold values for different dates and regions when the statistics are beyond the normal range.

By subsetting thresholds variation constrained to the stability region, Landsat-8 RMSE statistics are drastically improved. As illustrated in Table 3, mean RMSE is reduced by 23.72%, 37.38%, and 7.31% for *n* = (1,3,7), respectively. The CI_95%_ upper limit is reduced by 60.39%, 64.73%, and 0.88% for *n* = (1,3,7), respectively.

Sentinel-2 RMSE statistics improvement is not so elastic compared to Landsat-8. As illustrated in Table 3, mean RMSE is reduced by 24.88%, and 1.59% for *n* = (1,7), respectively, and increases 2.32% for *n* = (3). The CI_95%_ upper limit is reduced by 64.04%, 1.42% and 0.88% for *n* = (1,3,7), respectively.

Regarding the threshold variation, Landsat-8 thresholds varied 42.4% in the range of −0.33 < T < −0.19, and Sentinel-2 thresholds varied 11.1%. This result is superior to the limit of 10% considered by Herndon [20] as the possible range of optimal threshold value variation. However, this must be observed with a caveat since the performance metric used is different, and the study area is very constrained.

As illustrated in Table 4, there is a slightly generalized precision gain for both satellite dataset imagery. For *n* = (3) precision is increased for all instances: 0.5 m, 1.0 m, 1.25 m, and 2.0 m. After restricting the threshold variation for Sentinel-2, ~99% of the errors are lower than 1 m, and ~100% of RMSE are lower than 1.25 m.

### 3.3. The Non-Optimistic Error

Table 2 illustrates that the mean RMSE* increases as the number of images employed in threshold optimization grows. This can be explained due to inter-scenes reflectance variability, making it difficult to adjust a single threshold over a higher image dataset.

By comparing the ratio between mean RMSE (Table 3) and RMSE* (Table 2), it can be noticed how optimistic the error estimator is in the calibration step, depending on the number of images employed. Optimization that uses a single image (*n* = 1) chosen by chance can produce overfitted thresholds, too specialized in extracting water features from a specific scene, but when applied in another image, can lead to errors 20.8 times higher than expected.

Table 3 also shows that using three scenes (*n* = 3) in the optimization, the ratio RMSE/RMSE* is significantly reduced, indicating that the calibration and test sample variability become closer.

Conversely, the ratio RMSE/RMSE* less than 1, when *n* = (7), illustrated in Table 3, suggests that the same information employed in threshold calibration is used in test samples. This is because we did not employ pure cross-validation but a mimicked approach. Therefore, as the sample size (*n*) increases, considering the small Landsat-8 and Sentinel-2 imagery employed in this experiment, the probability of using the same information also grows.

Our results indicate that using a sample size greater than three (*n* > 3) to optimize single thresholds (T*) leads to non-overfitted models and are more suitable for multi-temporal analysis once it creates a good balance between bias and variance: a desirable characteristic of predictive models [33].

RMSE [T_i_ ≤ T ≤ T_f_] is the mean square error statistic after subsetting the sample. Landsat-8 thresholds were subsets to [−0.33 ≤ T ≤ −0.19] and Sentinel-2 thresholds were subsets to [−0.36 ≤ T ≤ −0.32] limits, derived from Figure 6 analysis.

### 3.4. Comparison between Otsu and Single Optimized Threshold

By comparing optimized thresholds (Table 2) to OTSU values (Table 5), one can notice that automated threshold values calculated by the OTSU method are higher than the optimized ones.

Mean RMSE values from OTSU (Table 6) are also higher than optimized ones (Table 3), however, maximum RMSE values from OTSU are lower than the optimized ones. When comparing results constrained in the stability region, mean and maximum RMSE values from optimized thresholds are dramatically superior to OTSU.

These results follow Ji and Wylie [18] and Herndon [20], that recommend the adjustment on the actual situation and calibrated to the local environment, aiming to improve performance.

### 3.5. Ensemble as Alternative to Single Threshold Approach

Our primary objective was to investigate uncertainty concerning the single threshold method and support its best choice. It was possible to identify a more stable region where thresholds can vary without impacting accuracy negatively. We investigated how better a model performs in the stability region and evaluated ways to securely set threshold values in the stability region using optimized threshold statistics, aiming to provide elements to support threshold choice.

Nevertheless, as pointed out, single thresholds can also be classified as a machine learning model, more specifically, a one-level decision tree. This understanding brings a new insight into results and the applied methodology, helping construct an easy-to-to alternative to the single threshold.

Usually, the decision tree model is sensitive to trained data [38], tending to produce unstable results with high variance [33]. Bagging or bootstrap aggregation [39] was one of the first ensemble technics developed. It combines the prediction of multiple models fitted over bootstrap samples, which are averaged to result in the bagged model’s prediction [40]

Mainly applied to decision trees, bagging can reduce the variance of unstable models and give a substantial gain in accuracy, making the final prediction more stable [39,40]. It occurs because averaging reduces variance and keeps bias unchanged [33]. Therefore, the power of bagging consists of combining models that have different perspectives on data [38].

As an alternative to the single threshold setting in the stability region, a bootstrap aggregation model would be constructed by ensembling optimized thresholds (T*) shown in Table 2 or using the constrained interval.

It is important to highlight the RMSE values shown in Table 3 do not correspond to bagged prediction accuracy once it was calculated as the mean of 500 RMSE values. Moreover, bagging averaging prediction must consider the number of models trained in bootstrap samples (in our experiment N = 20), and WL estimations (*Yc*) should be averaged before RMSE calculation.

Another observation is that bagging is suitable for models of high variance. Hence, the recommendation of employing more than three image scenes in optimization (discussed in Section 3.3) requires more investigations before constructing the ensemble model.

## 4. Conclusions

Using a single threshold to extract water features in multitemporal analysis leads to unavoidable errors that can be minimized by setting values constrained to specific limits, in which errors remain stable, and threshold values can vary without affect causing loss of accuracy.

The accuracy stability region can be identified by employing a cross-validation approach or using the whole imagery dataset. It happens because the limits of the accuracy stability region do not depend on the sample size employed in optimization. Conversely, the cross-validation approach is more time-consuming, requires more computational effort, and is not easy to use. The results also showed that the mean statistics of optimized thresholds values, even when carried out over singles scenes, can take thresholds close to the lower limit of the stability region.

This paper also examined the relationship between the number of scenes employed in single threshold optimization and the non-optimistic error. The results showed that the number of images plays a critical role in the model accuracy. More than three scenes randomly selected are always recommended. If a large number of free-of-cloud images is available, cross-validation (not mimicked) can be employed to provide this information more precisely.

The single threshold optimized by field observations was superior to the automated water extraction by the OTSU method. The performance of Sentinel-2 was superior to Landsat-8 imagery. Additionally, the OTSU method could not extract the maximum potential of Sentinel-2 imagery compared to the threshold method. Further research aims to investigate whether these results will be maintained by varying factors, such as spatial resolution, water index from different spectral bands, and other surface correction algorithms.

This paper showed that non-optimistic errors involved in applying a single threshold over MNDWI might not be compatible with the purpose of operational monitoring, even when employing a high-resolution digital elevation model. Considering that the single threshold method is a one-level decision tree, better accuracy would be expected by ensembling multiple optimized thresholds values.

Future research may use high-resolution digital elevation models associated with observed water levels and their respective contours levels to identify water pixels with high confidence and use them as training data. Experiments carried out at subpixel scale by DeVries [32] and Jones [31] indicate that machine learning algorithms and empirical decision rules, fitted over satellite bands, water, and vegetation spectral indexes, can aggregate more flexibility, improving water classification.

## Figures and Tables

**Table 1:** Filled gaps in observed water level data.

Satellite	Image Acquisition Date	CDAY	ΔT (Days)	OWL_CDAY (m)	Filled OWL (m)
Landsat-8	14 April 2013	16 April 2013	2	424.31	424.35
	13 September 2016	5 October 2016	22	417.62	416.97
	29 September 2016	5 October 2016	6	417.62	417.44
	15 October 2016	5 October 2016	10	417.62	417.28
	2 December 2016	21 December 2016	19	415.00	415.65
	4 February 2017	10 February 2017	6	414.31	414.46
	12 June 2017	31 July 2017	49	413.60	413.16
	15 August 2017	31 July 2017	15	413.60	413.44
	2 October 2017	31 October 2017	29	412.59	412.91
	5 December 2017	20 November 2017	15	412.29	412.84
	6 January 2018	19 February 2018	44	415.64	414.02
	2 June 2019	3 June 2019	1	420.61	420.62
Sentinel-2	1 January 2019	31 December 2018	1	419,18	419,17
	2 November 2019	3 November 2019	1	419.52	419.53
	17 November 2019	18 November 2019	1	419.38	419.39
	12 December 2019	13 December 2019	1	419.30	419.32
	10 April 2020	17 April 2020	7	431.82	431.43
	4 July 2020	3 July 2020	1	432.42	432.41

Note: CDAY: closest day with available OWL. ΔT: elapsed time in days between images’ acquisition date and the CDAY. OWL_CDAY: observed water level at the CDAY. Filled OWL: Filled observed water levels gaps by linear interpolation.

**Table 2:** Accuracy and threshold optimized statistics.

MNDWI	*n*	T*					RMSE*				
		Min.	Max.	Mean	SD	CV	Min.	Max.	Mean	SD	CV
Landsat-8	1	−0.440	−0.190	−0.325	0.097	−0.299	0.000	0.208	0.050	0.064	1.281
	3	−0.430	−0.190	−0.335	0.062	−0.177	0.005	1.368	0.611	0.478	0.783
	7	−0.410	−0.300	−0.328	0.033	−0.101	0.073	1.354	0.848	0.355	0.418
Sentinel-2	1	−0.393	−0.320	−0.365	0.030	−0.082	0.001	0.093	0.022	0.021	0.955
	3	−0.387	−0.323	−0.353	0.023	−0.065	0.040	0.501	0.227	0.130	0.573
	7	−0.383	−0.330	−0.351	0.017	−0.048	0.091	0.466	0.315	0.109	0.346

Note: T* is the optimized thresholds and RMSE* is the accuracy by varying the size sample (n) and imagery collection. SD: standard deviation. CV: coefficient of variation.

**Table 3:** Accuracy comparison after constraining threshold variation in a range of RMSE stability.

MNDWI	*n*	RMSE				RMSE/ RMSE*	RMSE [T_i_ ≤ T≤ T_f_]			
		Min.	Max.	Mean	CI_95%_		Min.	Max.	Mean	CI_95%_
Landsat-8	1	0.002	13.783	1.041	[0.027;6.039]	20.8	0.014	2.574	0.794	[0.063;2.392]
	3	0.000	14.108	1.043	[0.021;6.427]	1.71	0.041	2.362	0.653	[0.046;2.267]
	7	0.012	8.908	0.746	[0.067;2.263]	0.88	0.088	2.290	0.691	[0.100;2.243]
Sentinel-2	1	0.001	4.813	0.422	[0.014;2.392]	19.2	0.023	1.025	0.317	[0.044;0.860]
	3	0.001	3.693	0.301	[0.023;0.915]	1.33	0.000	1.257	0.308	[0.038;0.902]
	7	0.001	3.067	0.314	[0.023;0.910]	0.99	0.003	1.052	0.309	[0.044;0.902]

Note: RMSE is the non-optimistic error obtained from cross-validation; RMSE* is the mean optimized statistic (Table 2).

**Table 4:** RMSE cumulative empirical probability function obtained by mimicked cross-validation.

MNDWI	*n*	P (RMSE < X) × 100				P (RMSE [T_i_ < T < T_f_] < X) × 100			
		<0.5 m	<1.0 m	<1.25 m	<2.0 m	<0.5 m	<1.0 m	<1.25 m	<2.0 m
Landsat-8	1	46.6	75.2	77.0	85.8	38.7	79.3	80.5	88.0
	3	59.2	73.0	75.8	85.4	61.1	83.3	84.9	90.5
	7	59.6	78.8	79.4	88.0	61.2	81.6	81.6	87.3
Sentinel-2	1	76.0	91.2	93.4	96.0	84.3	99.4	100	100
	3	86.4	98.0	99.2	99.6	87.1	98.9	99.7	100
	7	96.0	98.8	99.2	99.8	83.4	99.5	100	100

Note: for the images MNDWI Landsat-8 [−0.33 ≤ T ≤ −0.19] and MNDWI Sentinel-2 [−0.36 ≤ T ≤ −0.32].

**Table 5:** OTSU thresholds statistics.

MNDWI	OTSU Thresholds					
	Min.	Max.	Mean	Median	Q_1_	Q_3_
Landsat-8	−0.363	0.074	−0.060	−0.051	−0.094	−0.008
Sentinel-2	−0.215	0.082	−0.116	−0.121	−0.154	−0.094

Note: Q_1_—first quartile; Q_3_—third quartile.

**Table 6:** Accuracy statistics from OTSU method.

MNDWI	OTSU Thresholds					
	Min.	Max.	Mean	Median	Q_1_	Q_3_
Landsat-8	0.168	9.924	1.751	1.143	1.046	1.602
Sentinel-2	0.812	1.791	1.197	1.115	0.991	1.352

Note: Q_1_—first quartile; Q_3_—third quartile.
